# Elevated atmospheric CO_2_ concentrations caused a shift of the metabolically active microbiome in vineyard soil

**DOI:** 10.1186/s12866-023-02781-5

**Published:** 2023-02-21

**Authors:** David Rosado-Porto, Stefan Ratering, Yvette Wohlfahrt, Bellinda Schneider, Andrea Glatt, Sylvia Schnell

**Affiliations:** 1 Institute of Applied Microbiology, Justus Liebig University, 35392 Giessen, Germany; 2 Faculty of Basic and Biomedical Sciences, Simón Bolívar University, 080002 Barranquilla, Colombia; 3 Department of General and Organic Viticulture, Hochschule Geisenheim University, Von-Lade-Strasse 1, 65366 Geisenheim, Germany

**Keywords:** Active soil bacterial community, Carbon cycle, Nitrogen cycle, Vineyard, rRNA, mRNA quantification, CO_2_, FACE

## Abstract

**Background:**

Elevated carbon dioxide concentrations (eCO_2_), one of the main causes of climate change, have several consequences for both vine and cover crops in vineyards and potentially also for the soil microbiome. Hence soil samples were taken from a vineyard free-air CO_2_ enrichment (VineyardFACE) study in Geisenheim and examined for possible changes in the soil active bacterial composition (cDNA of 16S rRNA) using a metabarcoding approach. Soil samples were taken from the areas between the rows of vines with and without cover cropping from plots exposed to either eCO_2_ or ambient CO_2_ (aCO_2_).

**Results:**

Diversity indices and redundancy analysis (RDA) demonstrated that eCO_2_ changed the active soil bacterial diversity in grapevine soil with cover crops (*p*-value 0.007). In contrast, the bacterial composition in bare soil was unaffected. In addition, the microbial soil respiration (*p*-values 0.04—0.003) and the ammonium concentration (*p*-value 0.003) were significantly different in the samples where cover crops were present and exposed to eCO_2_. Moreover, under eCO_2_ conditions, qPCR results showed a significant decrease in 16S rRNA copy numbers and transcripts for enzymes involved in N_2_ fixation and NO_2_^−^ reduction were observed using qPCR. Co-occurrence analysis revealed a shift in the number, strength, and patterns of microbial interactions under eCO_2_ conditions, mainly represented by a reduction in the number of interacting ASVs and the number of interactions.

**Conclusions:**

The results of this study demonstrate that eCO_2_ concentrations changed the active soil bacterial composition, which could have future influence on both soil properties and wine quality.

**Supplementary Information:**

The online version contains supplementary material available at 10.1186/s12866-023-02781-5.

## Background

Vineyards are important economic and agricultural ecosystems. According to the “Deutsche Wein Statistik” in 2017, the total land used for vineyards worldwide are 7.654 million hectares of which 3.312 million are in the European Union and 0.102 million in Germany. As a perennial culture, grapevines (*Vitis vinifera* L.) grow in a complex and dynamic ecosystem, where climate, soil, microorganisms, and management practices are key factors for plant health, productivity, and wine quality. These complex interactions in the local growing area, together with the viticulture and enological techniques, lead to the wine's unique taste (the terroir). Therefore, altering factors in this balance may alter the terroir and lead to changes in consumer acceptance and profitability. The increasing CO_2_ concentrations and the resulting climate change could both conceivably influence plant physiology and microbial ecosystems in vineyards.

Elevated CO_2_ concentrations can modulate the transcriptional and metabolic profiles and the stress responses of C3 plants and consequently affect their vegetative and reproductive development. For example, Wohlfahrt et al. [1] reported that under eCO_2_ conditions, the varieties Riesling and Cabernet Sauvignon presented higher net photosynthesis rates of 32% and 28%, respectively. Similarly, vines were reported to show higher net photosynthetic rates combined with reduced water availability under eCO_2_ and elevated temperature conditions [2, 3]. Additionally, eCO_2_ has been demonstrated to affect berry size and must properties as a result of increases in lateral leaf area, summer pruning fresh weight and yield; and altering malic and tartaric acids concentrations respectively [4, 5]. Furthermore, eCO_2_ concentrations expected in the future could influence interactions between plants and herbivorous insects. For example, Reineke et al. [6], showed that the transcriptional patterns of vine plants in response to the herbivorous insect *Lobesia botrana* differed under eCO_2_ and aCO_2_ conditions.

Different methodologies have been used to assess the effects of elevated atmospheric CO_2_ levels on soil ecosystems, including free-air CO_2_ enrichment (FACE). A FACE study facility on vines was established in the wine-growing region Rheingau by the Geisenheim University in 2014. Since then, several studies have been conducted in this facility examining the effects of expected future eCO_2_ concentrations on different aspects of grapevine physiology, yield efficiency, grape composition, and ecology [1, 5–7].

Various studies have examined the grapevine microbiome under normal atmospheric conditions [8–10]. These investigations have demonstrated differences between the microbial composition of the different parts of the vine plant and the surrounding soil microbiome, indicating a particular niche adaptation of distinct taxonomic groups to each plant component [8–10]. However, soil still plays an essential role as a major reservoir of microorganisms making up the vine microbiome [8–10]. Nerva et al. [11] described that pathogens associated with the chronic and complex wood disease known as ESCA (Black Measle) and grapevine trunk disease were more abundant in the soils of affected plants, indicating that the soil represents an essential source of inoculum. Likewise, other studies have established that independent of the growing region, rootstocks have a core microbiome that influences the taxonomy, structure, and microbial community in grapevine roots [12, 13]. Also, Liu et al.[14] showed that the fungal microbiome was influenced by grapevine habitat and plant development stage and the core microbiome members changed through a seasonal community succession.

eCO_2_ has been shown to increase the concentrations of sugars, amino acids, and organic acids in plant root exudates and consequently directly influences the soil microbiome structure and composition [15, 16]. Several studies have shown that the structure and function of the soil microbiome changed due to eCO_2_ conditions [17–21]. Moreover, larger carbon inputs under eCO_2_ may increase the microbial nitrogen demand, and thus the nitrogen dynamics are more likely to change under eCO_2_ [22]. Nevertheless, eCO_2_ effects on the microbiome of vineyard soil have not been studied to date.

Therefore, the present study assessed the effects of eCO_2_ on the bacterial composition of a vineyard soil planted with two *Vitis vinifera* cultivars in samples taken between the vine rows with (green) and without (open) cover crops. It was hypothesized that the soil bacterial composition and the abundances of N cycle transcripts were significantly affected by the eCO_2_ treatment.

The aims of the work were: i) to assess the effect of expected eCO_2_ concentrations in the mid term on active soil bacterial composition through an rRNA-based metabarcoding approach and ii) to study how changes in soil microbiome are connected to environmental variables.

## Results

### Ion torrent sequencing

A total of 3,903,289 raw sequences were obtained. After demultiplexing, sequences were assigned to each sample, with sequence counts in each sample ranging from 135,651 to 34,214. After quality control, denoising, sequence dereplication, and chimera filtering with DADA2 software, 2,010,680 sequences were removed, resulting in 1,892,609 non-chimeric sequences that were grouped into 10,708 amplicon sequence variants (ASVs) with a 99% similarity. Later, sequences belonging to chloroplast and mitochondria were removed, resulting in 10,583 ASVs from 1,887,273 total sequences.

### Soil microbial diversity

**Fig. 1:**
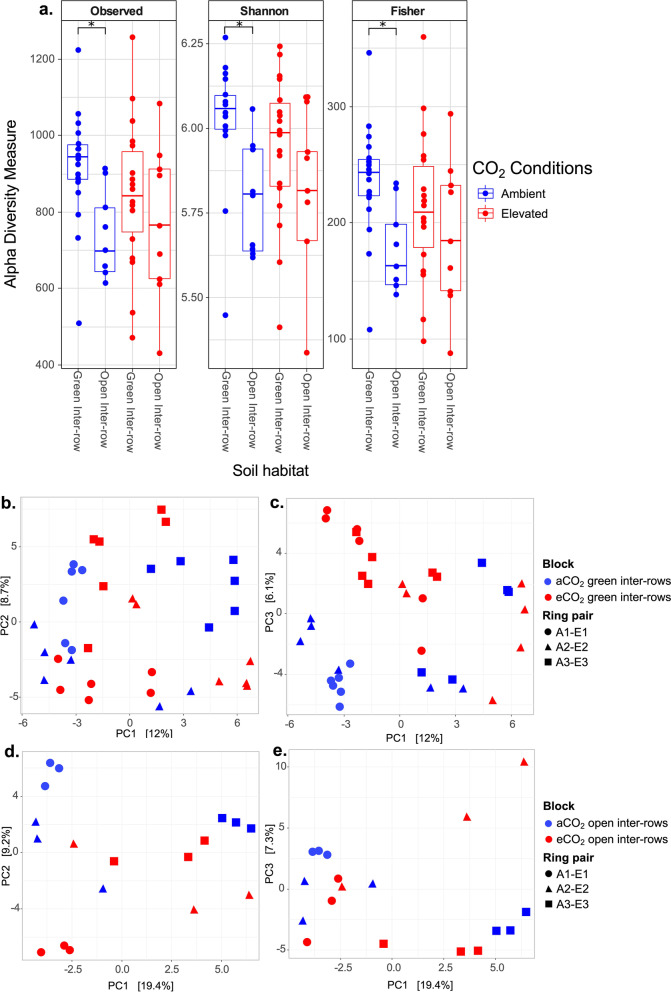
Diversity analysis of VineyardFACE experiment. **a** Alpha diversity metrics. aCO_2_, ambient CO_2_ conditions; eCO_2_, elevated CO_2_ conditions. * *p* < 0.05. **b, c** Principal Components Analysis (PCA) calculated based on Aitchison community dissimilarity distance matrix of axis 1–2 (left) and axis 1–3 (right) of green inter-rows from ambient and elevated CO_2_ rings, **d, e** Principal Components Analysis (PCA) calculated based on Aitchison community dissimilarity distance matrix of axis 1–2 (left) and axis 1–3 (right) of open inter-rows from ambient and elevated CO_2_ rings. A, ambient CO_2_ rings; E, elevated CO_2_ rings; aCO_2_, ambient CO_2_ conditions; eCO_2_, elevated CO_2_ conditions

In the soil from the Geisenheim VineyardFACE experiment, the bacterial diversity of the active bacterial component differed under elevated atmospheric CO_2_ concentrations. Our results indicate that under ambient CO_2_ (aCO_2_) conditions, the soil samples from green inter-rows showed significantly higher alpha diversity values than the open samples, according to species richness (observed ASVs; *p*-value 0.014) and species diversity (Shannon’s diversity; *p*-value 0.0074 and Fisher's alpha parameter; *p*-value 0.014) (Fig. 1a). Nevertheless, in the eCO_2_ treatments there was no statistical difference between green and open inter-rows (Fig. 1a). The relative abundance of the ten most abundant taxa on phylum, class and family level is shown in S1, Fig. S1.8-S1.10). Furthermore, although alpha diversity values do not show significant differences between samples from the green and open inter-rows from ambient and elevated CO_2_ rings, a slight decrease in the values of the different alpha diversity metrics of green samples from elevated CO_2_ treatment was observed (Fig. 1a).

The evaluation of the beta diversity in the VineyardFACE experiment was performed by creating a distance matrix using the Aitchison distance and later ordinated using the Principal Components Analysis (PCA). Initially, the degree of dispersion within the six rings was analyzed, including assessing their distance to the centroid. The results indicated that each soil core's bacterial composition was considerably different from the others, even those within the treatment (S1, Fig. S1.1-S1.6, Tab. S1.1-S1.6). Additionally, the analysis of soil bacterial composition indicated that the factors ring, block, and row (green or open inter-rows) significantly influenced the bacterial composition according to the PERMANOVA test (*p*-value 0.001). Likewise, CO_2_ conditions also significantly affected the overall bacterial composition, although to a lesser extent (*p*-value 0.002). Additionally, green soil sample diversity from ambient and elevated CO_2_ treatments showed substantial statistical differences in beta diversity (*p*-value 0.001) (Fig. 1b, c). Moreover, the ring factor significantly influenced the differentiation of bacterial compositions of green soil samples under elevated and ambient CO_2_ concentrations (*p*-value 0.001).

On the other hand, the beta diversity of the bacterial composition´s from open samples from ambient and elevated CO_2_ treatments showed no statistically significant differences between these two soils (*p*-value 0.123) (Fig. 1d, e). However, the ring factor essentially influences the structure of bacterial compositions in the open soil samples (*p*-value 0.001).

### Effect of environmental factors on microbial community

**Fig. 2:**
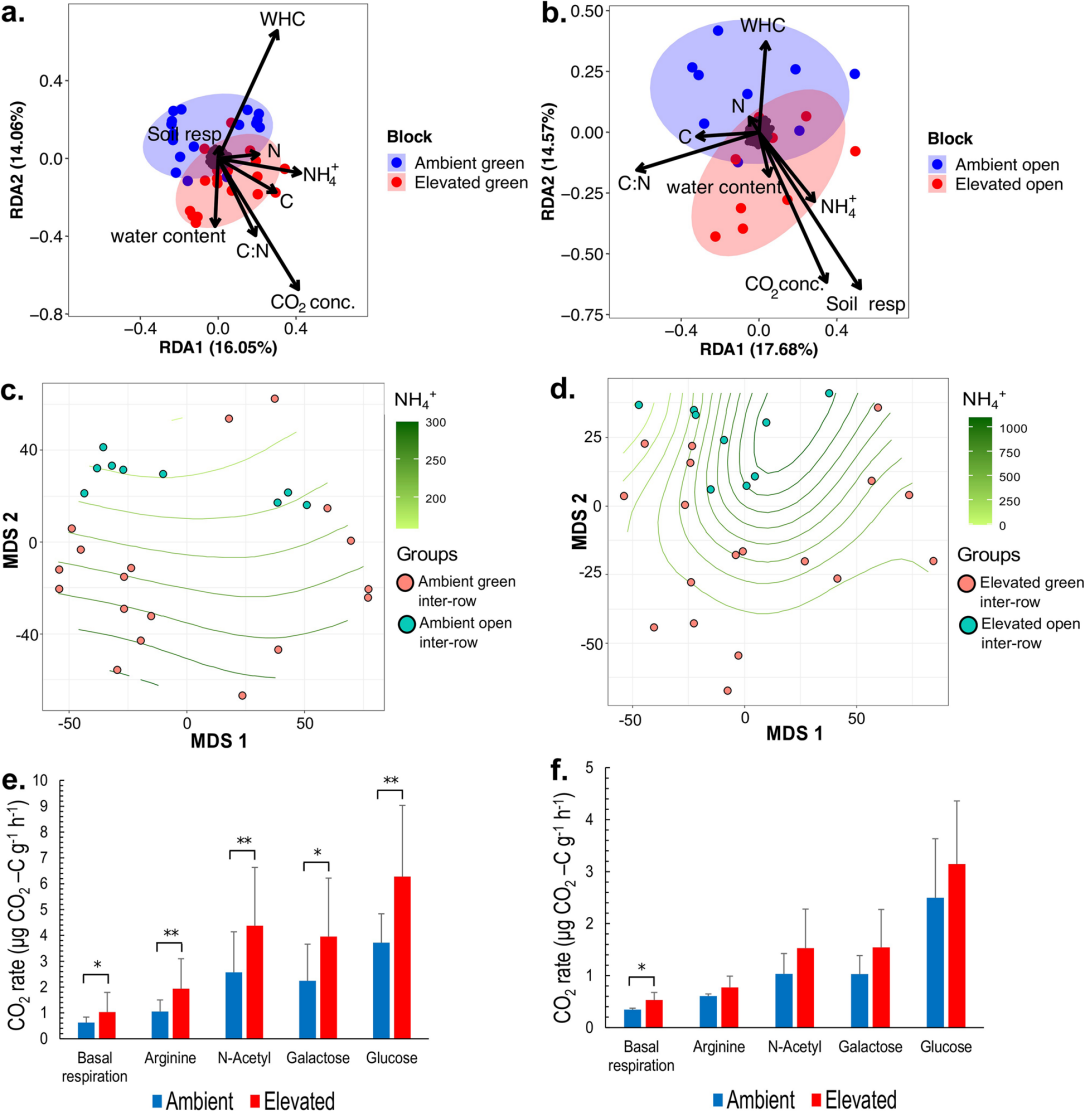
Environmental parameters effect on VineyardFACE experiment bacterial composition. **a**, **b** Redundancy Analysis (RDA) based on Aitchison community dissimilarity distance matrix of green inter-rows (left) and open inter-rows (right) from ambient (blue) and elevated (red) CO_2_ rings. WHC, Water holding capacity; CO_2_ Conc., CO_2_ concentration; Soil resp., Soil basal respiration; C, Total carbon concentration; N, Total nitrogen concentration; C:N, Carbon–nitrogen ratio; NH_4_^+^, Ammonium concentration. **c**, **d** Multidimensional scaling (MDS) with a grid of ammonium concentration expressed as (µM NH_4_*g^−1^), using Aitchison community dissimilarity distance matrix of green and open inter-rows from ambient CO_2_ rings (left), green and open inter-rows from elevated CO_2_ rings (right). **e**,** f** Soil microbial respiration expressed as CO_2_ production rate under the addition of different carbon substrates of green inter-rows from ambient and elevated CO_2_ rings (left), and open inter-rows from ambient and elevated CO_2_ rings (right). Error bars are expressed as variance of mean values

A redundancy analysis (RDA) was performed using a distance matrix based on the Aitchison distance to determine the effect of the different environmental factors that influence the bacterial composition structure of the VineyardFACE experiment. Results showed that eCO_2_ concentration significantly influenced the differentiation of the bacterial composition in green soil samples from ambient and elevated CO_2_ rings (*p*-value 0.007) (Table 1, Fig. 2a). Nevertheless, the effect of elevated CO_2_ on the differentiation of soil bacterial compositions of open samples was much weaker in comparison to that of the green samples and not statistically significant (*p*-value 0.102) (Table 1, Fig. 2b). Likewise, correlation analysis performed with ALDEx2 showed that ASVs belonging to the genera *Bradyrhizobium*, *Marmoricola*, *Nocardioides*, *Ilumatobacter*, and *Chthoniobacter* had significant positive correlations with environmental CO_2_ concentrations (Tab. S3.1, S3.2).

**Table 1:** Effect of environmental parameters on bacterial composition of green and open inter-rows soil samples

Environmental parameter	Green inter-rows	Open inter-rows
CO_2_ concentration	0.007 **	0.102
NH_4_^+^	0.015 *	0.035 *
Water holding capacity	0.003 **	0.240
Soil respiration	0.010 **	0.211
Water content	0.230	0.212
Total carbon	0.005 **	0.164
Total nitrogen	0.001 **	0.222
Carbon/Nitrogen ratio	0.686	0.260

Adjusted p-values of permutation test for redundancy analysis (RDA) based on Aitchison community dissimilarity distance matrix. ** *p* < 0.01, * *p* < 0.05

RDA showed that soil ammonium content had an important effect on the soil bacterial composition of the VineyardFACE experiment in both green (*p*-value 0.015) and open (*p*-value 0.035) inter-rows. Moreover, in the aCO_2_ treatments, green inter-row showed on average higher ammonium values than open areas (*p*-value 0.003) (Table 2, Fig. 2c). In contrast, in the eCO_2_ rings, open inter-rows showed higher ammonium concentrations than green inter-rows (*p*-value 0.025). Nevertheless, in general, the ammonium concentration was higher under elevated than ambient CO_2_ conditions (Table 2, Fig. 2d). In addition, some bacterial taxa presented significant correlations with soil ammonium content as an ASV from the uncultured family “*Entotheonellaceae*” and genus *Phenylobacterium*, which had negative and positive correlation coefficients, respectively.

**Table 2:** Average ammonium content of green and open inter-rows from ambient and elevated CO_2_ treatments

CO_2_ conditions	Green inter-rows NH_4_^+^ [µM g^−1^ DW soil]	Open inter-rows NH_4_^+^ [µM g^−1^ DW soil]	*p*-value
Ambient	245.66 ± 81.21	161.35 ± 39.3	0.003**
Elevated	370.44 ± 250.86	948.69 ± 628.71	0.025*

Error is expressed as standard deviation of mean values (*n* = 3). P-values significance codes are from a t-test for samples with unequal variances. Significance codes: ** *p* < 0.01, * *p* < 0.05

Likewise, water holding capacity (WHC), total nitrogen, and total carbon content are all factors that shaped bacterial composition differentiation of green inter-rows according to the permutation test of canonical axes in redundancy analysis (Table 1). In this regard, green inter-rows had significantly higher average values of these three environmental parameters than open inter-rows (S2), and several bacterial ASVs and genera showed significant correlations with each one of these parameters (S3).

The soil microbial respiration in the VineyardFACE experiment is a significant factor shaping the soil bacterial composition (*p*-value 0.034), showing higher soil respiration values in the eCO_2_ treatments. Furthermore, green inter-rows from eCO_2_ treatments showed significantly higher CO_2_ production in the basal respiration and with all examined substrates compared to aCO_2_ treatments (Fig. 2e). In contrast, in open inter-rows, soil respiration was higher in soils from eCO_2_ treatments, but only significantly higher in the basal respiration (*p*-value 0.02) (Fig. 2f). Additionally, soil microbial respiration was significantly higher in green compared to open inter-rows, in both eCO_2_ and aCO_2_ treatments; although, these differences were slightly higher under eCO_2_ conditions (Fig. S1.7, Tab. S1.7).

### Changes in microbial community composition of green inter-rows

**Fig. 3:**
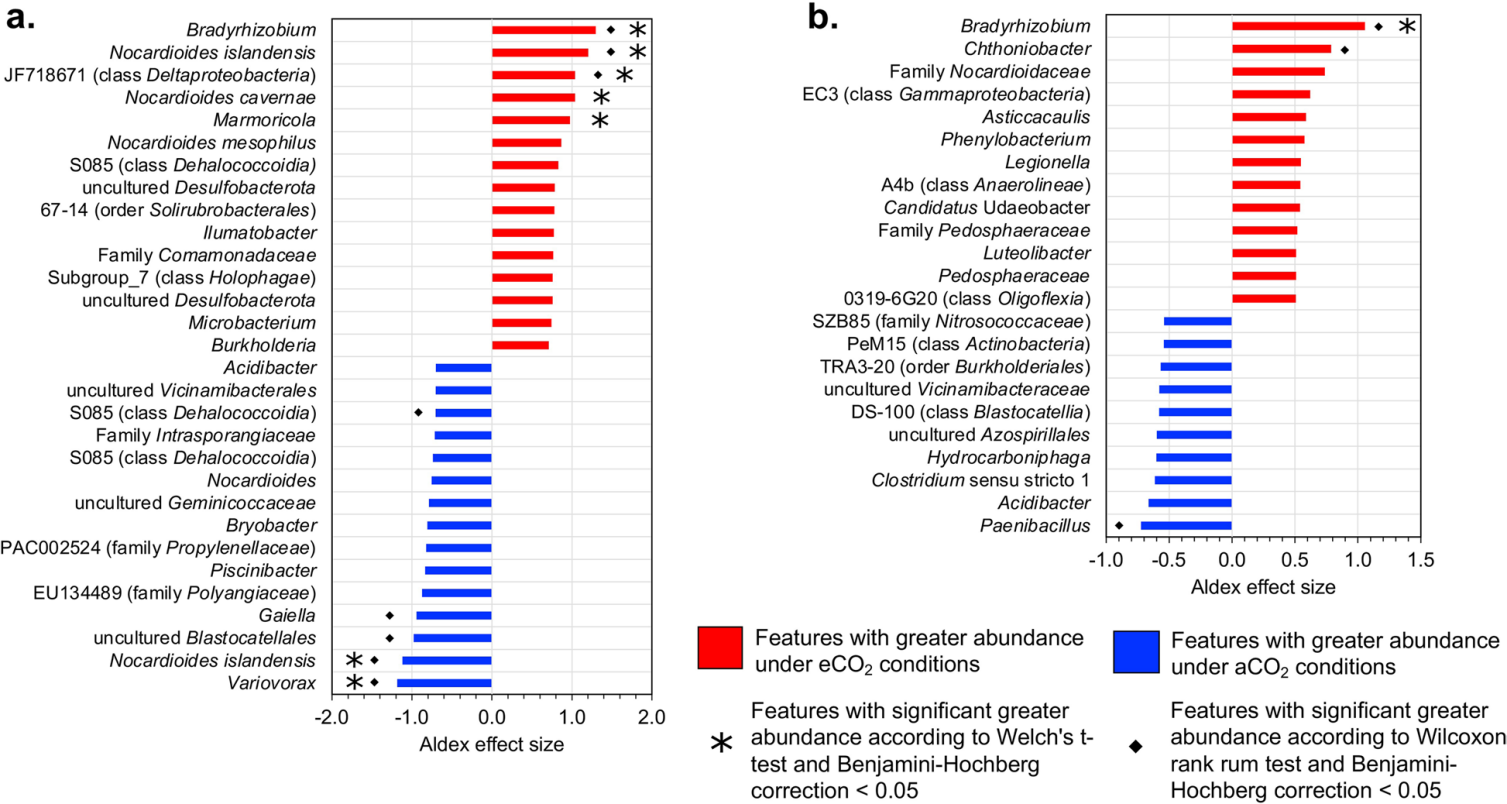
Differential abundances of core bacterial composition of green inter-rows soil under elevated and ambient CO_2_ of (**a**) Bacterial ASVs and (**b**) Bacterial genera. ALDEx2 results of features with an ALDEx2 effect size > 0.5 using centered log ratio (clr) transformation and the geometric mean abundance of all features

Differential abundance analysis confirmed that several core ASVs and genera showed changes in the green inter-rows soil samples under eCO_2_ conditions. In total, 44 ASVs and 13 genera were stimulated under eCO_2_ conditions. Among the highly stimulated ASVs in eCO_2_ treatments were *Bradyrhizobium*, *Marmoricola*, *Nocardioides mesophilus*, uncultured bacterium clone C10 (JF718671, class *Deltaproteobacteria*), *Nocardioides islandensis*, and *Nocardioides cavernae*, which presented ALDEx2 effect sizes between 0.86 and 1.29 and fold changes ranging from 1.75 to 366.32 (Figs. 3a, S4). Similarly, the core genera *Chthoniobacter*, *Asticcacaulis*, *Phenylobacterium*, *Legionella*, *Candidatus* Udaeobacter, *Luteolibacter*, and “*Pedosphaeraceae*” were positively stimulated under eCO_2_ concentrations, with ALDEx2 effect sizes between 0.78 and 0.5 and fold changes from 1.47 to 44.52 (Figs. 3b, S4). In contrast, 51 ASVs and 10 genera belonging to the core bacterial composition, decreased under eCO_2_ conditions. ALDEx2 results indicated that ASVs identified as *Variovorax*, *Nocardioides islandensis*, uncultured bacterium (EU192989, order *Acidobacteriales*), *Gaiella*, uncultured bacterium (EU134489 family “*Polyangiaceae*”), *Piscinibacter* and *Bryobacter* were the most affected by eCO_2_ in the green inter-rows. These ASVs showed ALDEx2 effect sizes between -0.8 and -1.18 and fold changes from 13.44 and 189.6 (Figs. 3a, S4). Additionally, the genera *Paenibacillus*, *Acidibacter*, *Clostridium* sensu stricto 1, *Hydrocarboniphaga*, uncultured bacterium (order *Rhodospirillales*), uncultured bacterium (DS-100, class *Blastocatellia*), uncultured bacterium (TRA3-20, order *Burkholderiales*), uncultured bacterium gene (clone SZB85, family “*Nitrosococcaceae*”) showed a reduction under eCO_2_ conditions with fold changes between 1.98 and 10 and ALDEx effect sizes ranging from -0.723 to -0.54 (Figs. 3b, S4).

### Co-occurrence analysis

**Fig. 4:**
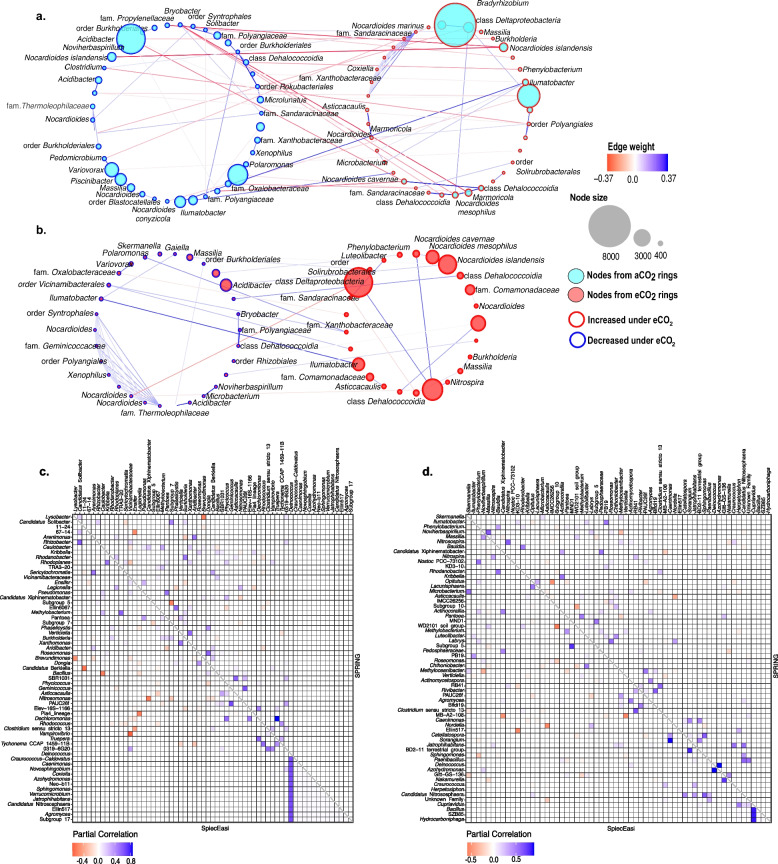
Co-occurrence analysis of features from green inter-rows. **a** Network analysis of core ASVs from aCO_2_ rings and **b** eCO_2_ rings. Features with an absolute ALDEx2 effect size > 0.5 were utilized for SpiecEasi analysis applying the Meinshausen & Bühlmann (mb) method with a number of subsamples of 50, n-lambda of 100 and lambda minimum ratio of 0.1; blue and red edges indicate positive and negative co-occurrence respectively; size of the nodes is proportional to the number of ASV reads. Partial correlation analysis of genera with an absolute ALDEx2 effect size > 0.1 from **c** aCO_2_ and **d** eCO_2_ green inter-rows using SpiecEasi and SPRING. SpiecEasi run applying the Meinshausen & Bühlmann (mb) method and SPRING with a modified centered log ratio (mclr). Both analyses utilized a number of subsamples of 99, a lambda minimum ratio of 0.1 and the Stability Approach to Regularization Selection (StARS) using co-occurences with a threshold of < -0.5 and > 0.5

Co-occurrence analysis results showed changes regarding co-occurrences among soil microorganisms under eCO_2_ concentrations. Networks from ASVs with ALDEx2 effect sizes greater than 0.5 showed a shift in the number, strength, and patterns of these microbial co-occurrences (Table 3). Under eCO_2_ conditions, there was a decrease in interacting ASVs and the number of co-occurrences. However, the average number of co-occurrences and the network density increased (Table 3, Fig. 4a). Also, negative co-occurrences decreased under eCO_2_ among these ASVs, with 26 (28.3%) negative co-occurrences in aCO_2_ green inter-rows compared to only 6 (8.8%) in the corresponding eCO_2_ samples. Moreover, most of the negative co-occurrences under aCO_2_ conditions occurred between nodes that were affected positively and negatively by the eCO_2_ (Fig. 4a). In contrast, under eCO_2_, interaction patterns changed, and those that did occur were mainly among ASVs that were negatively affected (Fig. 4b). Likewise, co-occurrence analyses performed with SpiecEasi and SPRING packages showed changes in co-occurrences of bacterial genera in the green inter-rows. There was no difference between these two conditions regarding co-occurring genera under aCO_2_ and eCO_2_. However, under eCO_2_, there were fewer co-occurrences (Table 3). Moreover, the number of positive co-occurrences greater than 0.25 is higher under eCO_2_ (8.7%) in comparison to aCO_2_ (4.6%). Furthermore, co-occurrence patterns indicated a shift in bacterial interactions due to eCO_2_. For example, the genus *Deinococcus* under aCO_2_ conditions showed positive partial correlations with 13 genera, among which were *Agromyces*, *Candidatus* Nitrososphaera, *Jatrophihabitans*, *Sphingomonas*, *Azohydromonas*, *Coxiella* and *Novosphingobium* (Fig. 4c). Nonetheless, most of these interaction patterns were no longer present under eCO_2_. In the case of the genus *Deinococcus* there was only one positive co-occurrence that with the genus *Azohydromonas* (Fig. 4d).

**Table 3:** Attributes of co-occurrence analysis from ASVs and genera belonging to green inter-rows

Co-occurrence attribute	aCO_2_ ASVs	eCO_2_ ASVs	aCO_2_ genera	eCO_2_ genera
Number of taxa	79	55	198	199
Number of co-occurrences	92	68	413	393
Average number of co-occurrences	2.33	5.7	4.17	3.95
Negative co-occurrences	26 (28.3%)	6 (8.8%)	132 (32.0%)	144 (36.6%)
Positive co-occurrences	66 (71.7%)	62 (91.2%)	281 (68.0%)	249 (63.4%)
Clustering coefficient	0.056	0.66	0.15	0.116
Network density	0.057	0.44	0.021	0.02

### cDNA Real time PCR

**Fig. 5:**
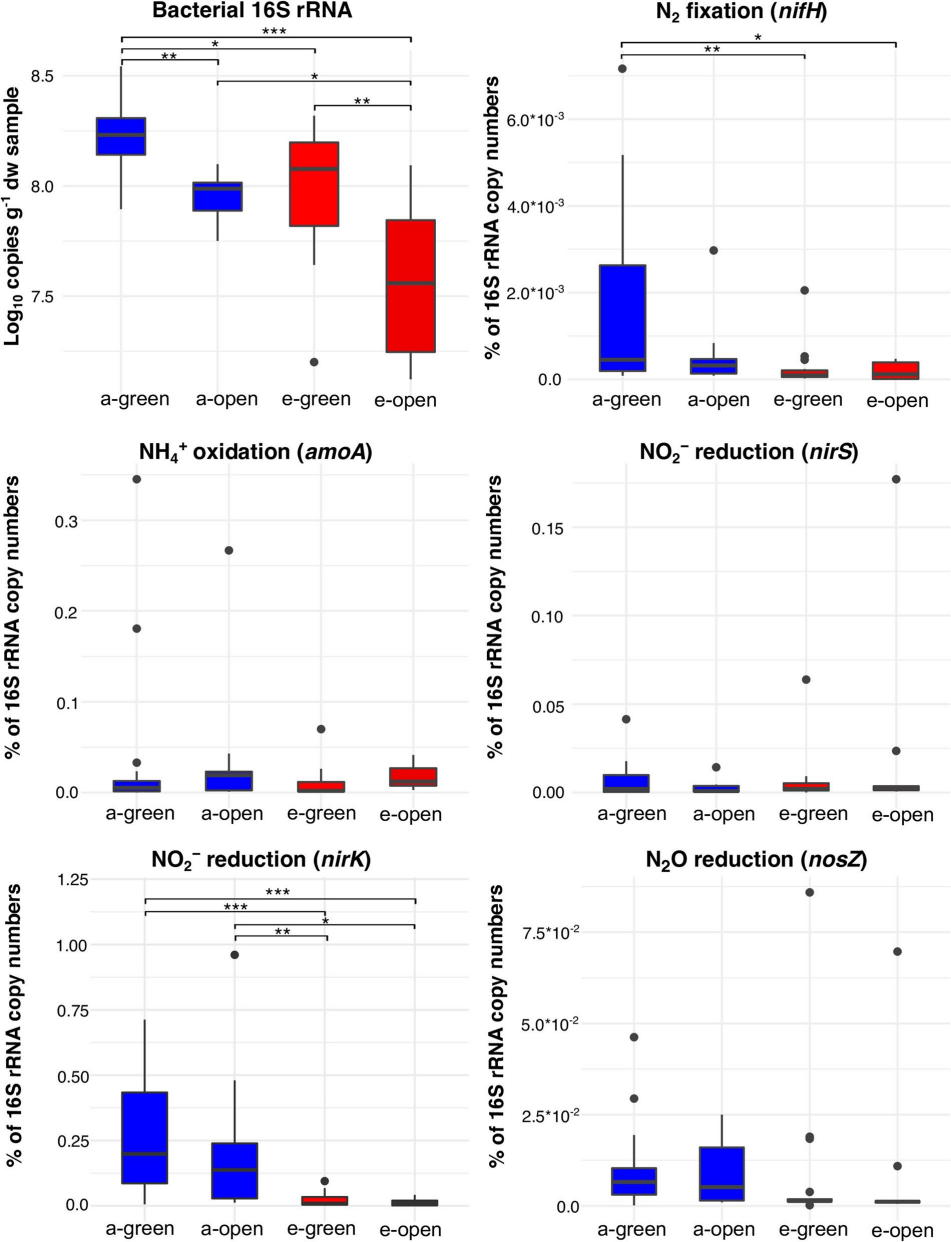
qPCR Boxplots of 16S rRNA, *nifH*, *amoA*, *nirS*, *nirK* and *nosZ* genes from aCO_2_ rings green inter-rows (a-green), aCO_2_ rings open inter-rows (a-open), eCO_2_ rings green inter-rows (e-green), eCO_2_ rings open inter-rows (e-open). Significance codes: *** *p* < 0.001, ** *p* < 0.01, * *p* < 0.05

The assessment of active bacteria through 16S rRNA quantification showed changes in the soil bacterial composition due to eCO_2_. In general, the number of active bacteria decreased under eCO_2_ conditions in both green and open inter-rows. On average aCO_2_ green inter-rows had significantly higher copy numbers per g dry weight of soil than the eCO_2_ samples (*p*-value 0.015) according to the Kruskal–Wallis test, about 36% more in aCO_2_ (1.81 ± 0.78*10^8^) in comparison to eCO_2_ (1.16 ± 0.56*10^8^). Also, aCO_2_ open inter-rows samples showed significant higher copies of 16S rRNA (8.93 ± 2.32*10^7^) compared to eCO_2_ (5.24 ± 4.03*10^7^) (*p*-value 0.047). Nonetheless, in both aCO_2_ and eCO_2_ treatments, green inter-rows showed higher values of active bacterial biomass compared to the open inter-rows (Fig. 5).

Similarly, to the 16S rRNA, the analysis of mRNA of functional genes involved in the nitrogen cycle indicated changes mainly in N_2_ fixation and the denitrification processes under eCO_2_ conditions (Fig. 5). The analysis of transcripts from the nitrogen fixation gene *nifH* showed a significant decrease under eCO_2_ in green inter-rows (*p*-value 0.007), with 84% fewer (2.75 ± 5.15*10^–4^) compared to aCO_2_ (1.69 ± 2.17*10^–3^) (Fig. 5). Likewise, the transcription of the NO_2_^−^ reduction gene *nirK* was affected negatively under eCO_2_ concentrations in both green and open inter-rows. eCO_2_ green inter-rows had an average of 2.09 ± 2.71*10^–2^ copies expressed as % of 16S rRNA copy numbers, compared to 3.11 ± 3.14*10^–1^ copies under aCO_2_ conditions, representing a decrease of 93%. Moreover, open inter-rows samples also showed higher values of *nirK* transcripts under aCO_2_ treatment (2.31 ± 3.12*10^–1^) compared to eCO_2_ (1.41 ± 1.55*10^–2^) (Fig. 5). Contrastingly, NO_2_^−^ reduction gene *nirS* transcripts did not show any differences between eCO_2_ and aCO_2_ conditions, neither between green inter-rows nor open inter-rows. Similarly, transcripts of the *nirS* gene, *amoA*, and *nosZ* genes involved in NH_4_^+^ oxidation and N_2_O reduction did not show any differences under the eCO_2_ conditions (Fig. 5).

## Discussion

### Microbiome structure and diversity

Grapevine (*Vitis vinifera* L. cv.) is one of the most extensively grown and economically important fruit crops, and the terroir of wines the main product of the grapes is influenced by physical (climate), biological (soil, microbiome, grape variety, fauna), viticultural and enological factors. It is well known that grapevines are particularly sensitive to changes in climatic conditions, among which increasing atmospheric CO_2_ concentrations has several consequences [1–3, 5, 6, 23, 24], However, it is not well known how climate change influences the microbes in which contribute to the terroir [25].

Our results demonstrated that increasing the atmospheric CO_2_ concentration altered the active soil microbiome structure in a vineyard, adding to the already reported effects on grapevine. However, RNA metabarcoding has its limitations mainly due to the RNA conversion to cDNA using of a reverse transcriptase which lacks proofreading activity, leading to point mutations in the cDNA [26]. In addition, reverse transcriptase can also lead to template switching, which can produce chimeric cDNA and the creation of shortened isoform sequences [27, 28]. However, in our study these limitations were minimized by using a Moloney murine leukemia virus reverse transcriptase (MMLV-RT) derivative combined with a *E. coli* DNA polymerase III ε subunit that lowers the reverse transcription error rate by threefold, and thereafter the resulting cDNA was amplified with a proofreading DNA polymerase which produced up to eightfold fewer errors [29].

Our data indicate that changes in soil bacterial composition occurred mainly in the green (vegetation covered) inter-rows soil samples from eCO_2_ treatments of the VineyardFACE experiment. Regarding alpha diversity, Observed ASVs, Shannon’s diversity, and Fisher's alpha parameter demonstrated that there are differences between green and open (no vegetation cover) inter-rows under aCO_2_ conditions; nonetheless, this difference was not found under eCO_2_ treatments. This indicates that under eCO_2_, a decrease in alpha diversity in the soil of green inter-rows occurred (Fig. 1a).

Soil bacterial composition structure and activity were highly affected by eCO_2_ in the VineyardFACE experiment, as indicated by our beta diversity results, (Fig. 1b-e). Increasing atmospheric CO_2_ was one of the environmental factors that significantly influenced the soil bacterial composition (Fig. 2a-b). Nevertheless, this change was only observable in the green inter-rows and not in the open ones, likely due to the presence of vegetation. Similar results have been reported in crop plants, such as wheat and soybean, in which eCO_2_ altered the structure of soil and rhizosphere bacterial compositions [30, 31]. Likewise, comparable results with significant changes have been described in the root and rhizosphere microbiota associated with *Phytolacca americana*, *Amaranthus cruentus*, and grassland ecosystems in response to eCO_2_ [17, 32, 33]. These changes could be a consequence of increased C and N inputs derived from the plant's increased rhizodeposition, which influences the composition and biomass of the soil bacterial community [34, 35].

Our data showed a significant increase of heterotrophic soil respiration on eCO_2_ soil samples, with average changes ranging from 1.65 to 1.85 fold, a sign of stimulated soil microbial activity. Nonetheless, our quantification of bacterial 16S rRNA through qPCR demonstrated a decline in bacterial abundance caused by eCO_2_ concentrations, which might be explained by an alteration of soil microbial structure in favour of fungal growth. This behavior has already been described in a chaparral ecosystem [36], a scrub-oak ecosystem [37], and a wheat-soybean agroecosystem [30], in which the ratio of fungi to bacteria was increased under eCO_2_ along with an enhancement of soil microbial heterotrophic respiration.

### eCO_2_ effect on the N cycle, changes in bacterial abundance and microbe-microbe interactions

**Fig. 6:**
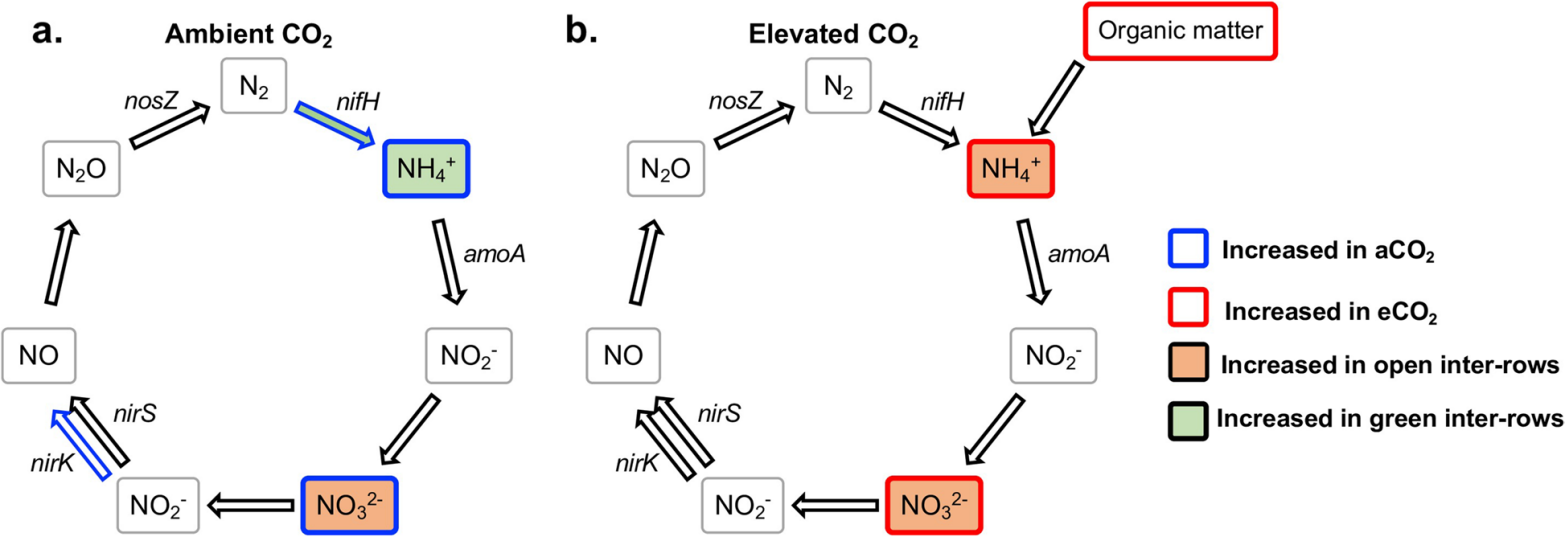
Model diagram of N cycle of VineyardFACE soil under (**a**) aCO_2_ conditions and (**b**) eCO_2_ conditions

Greater inputs of labile carbon under eCO_2_ conditions through root exudation increases the microbial nitrogen (N) demand, and consequently, nitrogen dynamics are likely to change under eCO_2_ conditions [22]. Several studies have investigated and shown the changes that genes, proteins, and microorganisms undergo due to eCO_2_ conditions, including an increase in numbers and/or activity [30, 38–42]. In contrast, some studies did not find any significant differences [43, 44]. In this sense, N_2_ fixation at eCO_2_ concentrations has generally been reported to increase in response to higher N demand due to excess C containing compounds [38, 39, 41, 42]. Nevertheless, our data did not indicate an increase in the N_2_ fixation in response to eCO_2_ but instead, nitrogenase *nifH* qPCR results demonstrated a decrease in N_2_ fixing activity in the green inter-rows under eCO_2_ conditions (Figs. 5 and 6). However, NH_4_^+^ concentrations were higher in eCO_2_ treatments compared to aCO_2_, indicating that although N_2_ fixation is downregulated in eCO_2_ conditions, microorganisms are obtaining NH_4_^+^ from other sources, probably from soil organic matter (SOM) (Fig. 6). Therefore, the increased supply of fresh plant-derived C into the soil matrix under eCO_2_ conditions may accelerate the decomposition of SOM and decrease soil C stocks [45, 46], a phenomenon known as “the priming effect”. Also, SOM pools contain significant physically and chemically protected N stocks. Therefore the priming effect is a response to the labile C supply by which microorganisms gain access to a reservoir of N to meet their enhanced N demand [47–49]. The aforementioned has been described by Müller et al. [22], who reported that under eCO_2_ conditions, the mineralization of labile organic N became more important, alongside an increment in the dissimilatory NO_3_^−^ reduction to NH_4_^+^ (DNRA) and in the immobilization of NH_4_^+^ and NO_3_^−^ [22]. This might explain why with some taxa (e.g. the genus *Phenylobacterium*) which have been reported to perform heterotrophic DNRA that were stimulated under eCO_2_ conditions there was a significant positive correlation with NH_4_^+^ concentrations (S3) [50].

Similar to *nifH*, it has been frequently reported that under eCO_2_ an increase of transcripts for denitrification genes *nirS*, *nirK*, and *nosZ* occurs [38, 39, 41]. However, our results did not show an increase in the abundance of mRNAs in these genes. In contrast, our data indicated an alteration of the denitrification process at the step of NO_2_^−^ reduction by decreasing *nirK* activity under eCO_2_ conditions (Figs. 5 and 6).

The alteration of N cycle-related gene transcripts seems to be associated with the decrease of certain bacterial taxa and the shift of the soil bacterial composition because of the selective pressures imposed by eCO_2_. Our co-occurrence data demonstrated a shift of bacterial taxa and a simplification of microbial interactions under eCO_2_ conditions. For example, the replacement of several N_2_ fixing bacteria with *nifH* genes such as *Microbacterium* [51, 52] and *Paenibacillus* [53–57] by the genus *Bradyrhizobium* [58–60] was observed in eCO_2_ treatments (S3). Likewise, the decreased abundance of *nirK* transcripts under eCO_2_ might be linked to the depletion of bacterial taxa such as *Noviherbaspirillum* [61], *Massilia* [62], and *Clostridium* sensu stricto 1 [63] which have been reported to perform NO_2_^−^ reduction and possess the gene responsible for this.

Network analysis results showed a strong positive co-occurrence between *Noviherbaspirillum* and *Microbacterium* under eCO_2_, which demonstrated that the depletion of these two genera is linked. Similarly, the co-occurrence cluster observed among ASVs negatively affected by eCO_2_ supports the idea that the increase of atmospheric CO_2_ concentrations disrupts soil microbial networks, and the depletion of certain bacterial taxa is entangled with the decrease of others. This cluster included ASVs belonging to genera *Xenophilus* and *Nocardioides* and ASVs from families *Geminicoccaceae* and *Thermoleophilaceae*. Additionally, partial correlation data displayed alterations in the co-occurrence patterns caused by eCO_2_, where taxa interacting with each other no longer exhibited these patterns. A good example is the genus *Deinococcus*, which at aCO_2_ showed positive interactions with 13 genera, but only retained its positive co-occurrence with the genus *Azohydromonas* at eCO_2_. This modification of interaction patterns is probably connected to changes in *nirK* mediated denitrification because the genus *Deinococcus* has also been reported to perform NO_2_^−^ reduction and possess this gene [64].

It has been reported in field experiments with tea seedlings (*Camellia sinensis* L. ‘Baihaozao’) that an increase in the quantity of *nirK* and *nosZ* genes was linked to the decline of N_2_O [65]. This might suggest that in the VineyardFACE experiment, the eCO_2_ might augment N_2_O emissions due to the alteration of the denitrification process reflected in the abundance of *nirK* gene transcripts. Moser et al. [66] also described that N_2_O emissions were 1.79-fold higher in the Giessen FACE under eCO_2_ conditions. Nonetheless, it is important to mention that in vineyards, N_2_O emissions depend on soil type, amount of fertilizers, and humidity, along with climate conditions [67], and those correlations with soil properties are likely to be highly system specific [68].

## Conclusions

Our results demonstrate that the increase in atmospheric CO_2_ concentrations has changed the structure and composition of the soil bacteria in the VineyardFACE experiment. This suggests that even with a relatively short period of eCO_2_ concentration in the VineyardFACE field, carbon cycle alterations have impacted the soil nitrogen cycle bacteria, producing a shift in diverse bacterial taxa. These soil bacterial composition changes could have more consequences on wine terroir and quality in the future. Nevertheless, additional analyses and time points will be necessary to assess alterations regarding the functional metatranscriptome due to eCO_2_ and its impact on wine production and grapevine health and productivity.

## Materials and methods

### Study site description

**Fig. 7:**
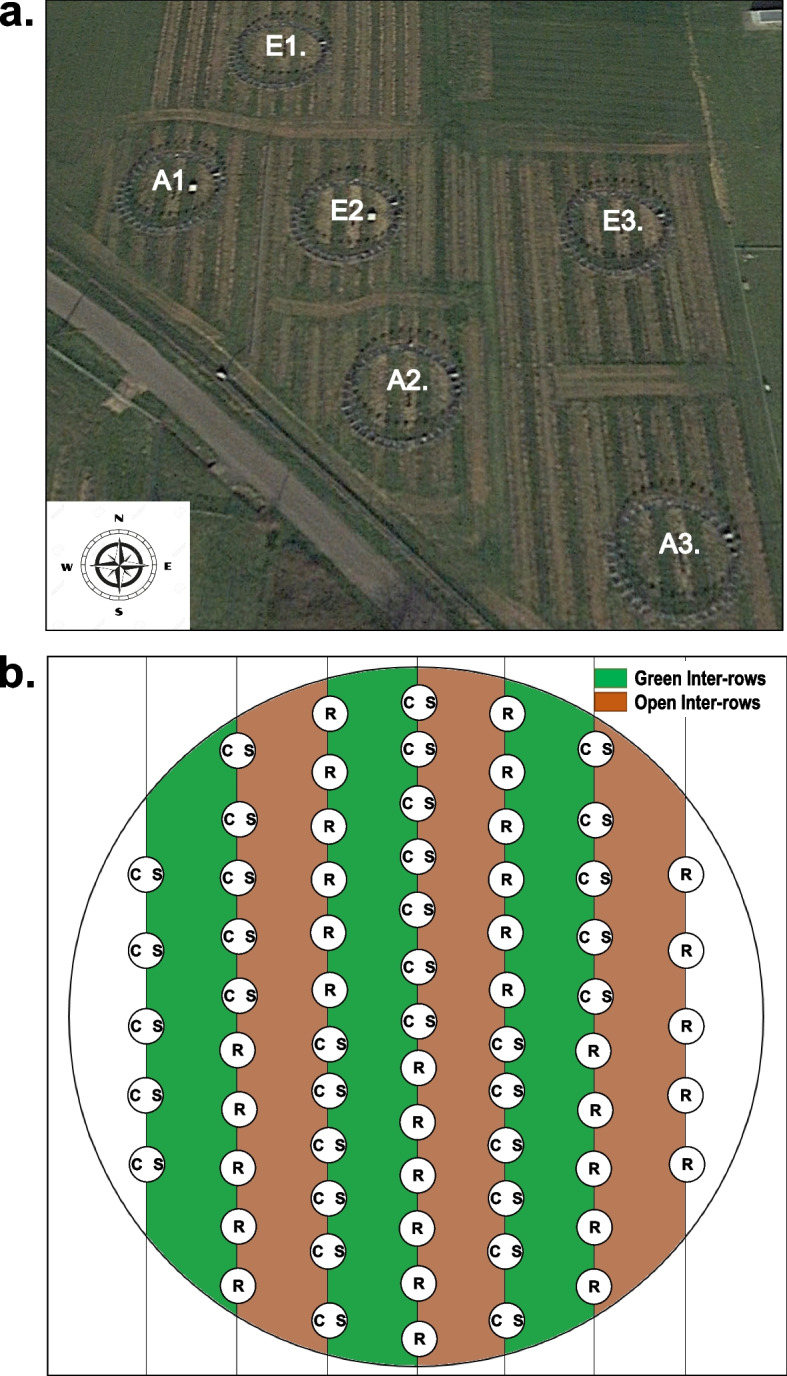
(**a**) Air view of VineyardFACE experimental site. E: elevated CO_2_ ring, A: ambient CO_2_ ring. Google Earth Pro Image (2021). (**b**) Design of a VineyardFACE-ring with the two grape varieties Riesling (R) and Cabernet Sauvignon (CS). The vertical lines represent the seven rows per ring of vine plants. Green-colored inter-rows represent the area within the ring with cover crop (green inter-rows) and brown-colored inter-rows represent the areas within the ring where the soil is periodically ploughed (open inter-rows)

The VineyardFACE facility is located at the Hochschule Geisenheim University, Germany (49°59′N, 7°57′E; 96 m above sea level) in the German wine-growing region Rheingau on the banks of the river Rhine. Geisenheim has a temperate oceanic climate (Köppen-Geiger classification: Cfb) with mild winters and warm summers. The mean annual temperature is 11.0 °C, and total annual precipitation averages 527.1 mm (long-term average from 1991 to 2020). The soil at the experimental site is characterized as low-carbonate loamy sand to sandy loam. The VineyardFACE experiment consists of three ring pairs (A1-E1, A2-E2, A3-E3), each with an inner diameter of 12 m, of which three are under elevated CO_2_ (eCO_2_; E1, E2, E3) and three under ambient CO_2_ (aCO_2_; A1, E2, E3) concentration. Within eCO_2_ rings, the air was enriched during daylight hours to approximately 18% above the ambient CO_2_. The average daily CO_2_ concentration of aCO_2_ and eCO_2_ treatments in June was 409.4 ± 8.6 and 483.2 ± 8.4 (means ± SD), respectively. Within VineyardFACE experiment rings, vines of *Vitis vinifera* L. cv. Riesling (clone 198–30 Gm) grafted on rootstock SO4 (clone 47 Gm) and cv. Cabernet Sauvignon (clone 170) grafted on rootstock 161–49 Couderc, respectively, were planted in April 2012 as one-year-old potted plants. Each ring contains seven rows of cv. Riesling and cv. Cabernet Sauvignon plants, which were planted alternately across a central divide. Vines were planted with a spacing of 0.9 m within rows and 1.8 m between rows, with a north–south orientation. Cover crops consisted of Freudenberger WB 130 mulch mixture III (10% *Lolium perenne*, 50% *Festuca rubra*, and 40% *Poa pratensis*) and was sowed to every second inter-row, identified in this work as green inter-rows; while every other second inter-row was plowed once in spring and was largely bare or covered with spontaneous vegetation identified in this work as open inter-rows (Fig. 7) [1, 6]

### Soil sampling and physico-chemical parameter measurements

Soil sampling was performed in June 2018. 50 ml sawed syringes (11 × 3 cm) were utilized, and 12 samples ~ 10 cm deep were taken from each ring, 6 from green inter-rows and 6 from open inter-rows. Half of the samples were taken to perform molecular biological and chemical analyses, and the other half to perform soil microbial respiration measurements. Green inter-rows soil cores were gently shaken by hand to remove loosely attached soil (bulk soil), and the soil that remained attached to the roots was considered rhizosphere soil. Soil cores from open inter-rows were only managed as bulk soil because no roots were present in them. Bulk and rhizosphere soils were sieved (< 2 mm) and stored at -80 °C for molecular biological analysis, at -20 °C for chemical analyses, and at 4 °C for soil microbial respiration analyses. Soil samples were classified in four blocks according to the CO_2_ conditions (ambient and elevated) and the inter-rows from where they were taken (green inter-row soil and open inter-row soil).

Ammonium concentrations were measured after soil extractions with 1 M KCl using a colorimetric assay [69]. Nitrate was extracted with deionized water, and the filtered supernatant was analyzed by ion chromatography (Sykam S5200 chromatograph, Sykam GmbH, Eresing, Germany) according to Bak et al. [70]. The water content, dry matter, and water holding capacity of soil samples were measured gravimetrically [71]. Carbon and nitrogen contents of the soil were measured by pyrolysis coupled to gas chromatography on an EA 1100 elemental analyzer (ThermoQuest, Milan, Italy) using a TCD detector by the Dumas method according to HBU (1996) [72] and VDLUFA (2012) method [73]. In each ring, CO_2_ concentration was recorded using an infrared gas analyzer (LI-840A CO_2_/H_2_O Analyzer, LI-COR Biosciences, Lincoln, NE, USA) mounted at 1.5 m height within the ring center.

Respiration analysis with the MicroResp™ system (James Hutton Ltd, Aberdeen, Scotland, UK) was performed following the protocol described by Campbell et al. [74]. Detection plates were prepared by mixing agar solution 3% and indicator solution (Cresol Red 12.5 µg ml^−1^, KCl 150 mM and NaHCO_3_ 2.5 mM) in a ration 1:2 (agar:indicator). Soil samples were weighed, added to deep well plates, and incubated for 3 days in a sealed box containing wet paper towels. Later, sterile distilled water and the substrates (L-Arginine, D-Galactose, D-Glucose, and N-Acetyl glucosamine) were added to each sample at a final concentration of 20 mM. The detection plate’s absorbance at time 0 was measured with a TECAN Infinite® M200 multimode Microplate Reader (Tecan Austria GmbH) at 570 nm, immediately assembled with the MicroResp™ seal (James Hutton Ltd) and the deep well plate and incubated for 6 h at 25 °C. Afterwards, the absorbance of the detection plate was read as described above. For the calculation of the CO_2_ production rate, data were normalized, and % CO_2_ was calculated with a previously prepared calibration curve using a spline fit with Origin Lab® software (OriginLabCorparation, Northhampton, USA). Later % CO_2_ values were converted to CO_2_ rate (µg CO_2_ – C g^−1^ DW soil h^−1^).

For chemical parameter results, measures of central tendency and dispersion were calculated. Ammonium, total carbon, total nitrogen, and carbon/nitrogen ratio differences among the four experimental blocks were assessed using a t-test for groups with similar variances. Differences in respiration results were calculated utilizing a t-test for samples with different variances using Microsoft Excel 2013.

### RNA extraction and reverse transcription

RNA extraction was performed following a modified protocol of Mettel et al. [75]. For the extraction, 0.3 – 0.5 g of soil were weighed in reaction tubes containing 100 mg of sterile zirconia beads, added with 700 µL TPM buffer (50 mM Tris–HCl (pH 5), 1.7% [wt/vol] polyvinylpyrrolidone, 20 mM MgCl_2_) and vortexed for 30 s. Cells were then disrupted in a cell mill MM200 (Retsch, Haan, Germany) for 2 min at a frequency of 30 Hz. Soil and cell debris were precipitated by centrifugation in a microcentrifuge (Heraeus Fresco, Thermo Fisher Scientific Inc., Waltham) for 5 min at 17,000 g and 4 °C, then the supernatant was transferred into a fresh reaction tube. Buffer PBL (770 µl, 5 mM Tris–HCl (pH 5), 5 mM Na_2_EDTA and 0.1% [wt/vol] sodium dodecyl sulfate) were added to the resulting soil pellet and the disruption process was performed again as described above. Both supernatants from the lysis processes were pooled in one reaction tube. The pooled supernatant was immediately extracted, initially with the addition of 500 µl of phenol/chloroform/isoamyl alcohol (25:24:1) and subsequently with chloroform/isoamyl alcohol (24:1). Afterwards, each time sample was centrifuged for 5 min at 17,000 g and 4 °C. The resulting upper aqueous phase was transferred to a new reaction tube, 800 µl of PEG solution was added (30% [wt/vol] polyethylene glycol 6000 and 1.6 M NaCl), incubated in ice for 30 min, and centrifuged for 30 min at 17,000 g and 4 °C. Subsequently, the DNA/RNA pellet was washed with 800 µl of ice-cold 75% ethanol, dried out and dissolved in 50 µl of nuclease-free water.

After extraction, samples were treated for DNA digestion with RNase-Free DNase Set (QIAGEN GmbH—Germany) according to the manufacturer’s instructions; DNase reaction was stopped with 10 µl of 50 mM EDTA. With the DNA-free RNA, a PCR was carried out using the universal 16S rRNA gene primers 27F (5’-AGAGTTTGATCMTGGATCMTGGCTCAG-3’) and 1492R (5’- GGTTACCTTGTTACGACTT-3’) [76, 77] and checked on agarose gel electrophoresis to verify the absence of remaining DNA in the samples. Subsequently, reverse transcription was performed utilizing an AccuScript High Fidelity 1st Strand cDNA Synthesis Kit (Agilent Technologies, Inc., Cedar Creek – Texas, USA) following manufacturer instructions.

### 16S rRNA Ion Torren sequencing and metagenomics analysis

The 16S rRNA gene hypervariable regions (V4&V5) were PCR amplified using the set of primers 520F (5’-AYTGGGYDTAAAGNG-3’) [78] and 907R (5’-CCGTCAATTCMTTTRAGTTT-3’) [79] and PCRs and sequencing by Ion Torrent technique were carried out according to the protocol described by Kaplan et al. [80]. Ion Torrent sequencing output was analyzed using QIIME2 version 2020.6 [81]. First, sequences were demultiplexed with the QIIME cutadapt command [82] using a barcode error rate of 0 and assigned to specific samples by corresponding barcodes. Later, quality control, denoising, sequence dereplication, and chimera filtering were performed using DADA2 software [83]. The first 15 nucleotides were trimmed, and sequences were truncated at a position of 320 nucleotides. Amplicon Sequence Variants (ASV) generated with DADA2 were taxonomically affiliated with a trained fitted classifier [84, 85] based on the SILVA 138 database [86, 87].

### Diversity and differential abundance analyses

Alpha and Beta diversity analyses were performed using R studio software 1.1.419, R packages Phyloseq 1.28.0 [88], and Vegan 2.4–6 [89]. For alpha diversity assessment, rarefaction was applied, and diversity indices (Observed ASVs, Shannon’s diversity, and Fisher's alpha parameter) were calculated and compared between CO_2_ conditions and soil habitats using the Wilcoxon test [90] with the Bonferroni correction method through 999 permutations. For non-constrained beta diversity analyses, data were transformed using the centered log ratio (clr) method [91, 92], using the R package Microbiome version 1.8.0 [93]. Later, community distance matrices were created using the Aitchison distance [91, 92] and visualized using principal components analysis (PCA) [94]. Statistical differences among blocks, rings, CO_2_ conditions, and ring plus soil habitats were assessed by a Permutational Multivariate Analysis of Variance (PERMANOVA) using the Adonis method and employing 999 permutations with the R package Vegan version 2.4–6 [95]. Additionally, the degree of dispersion of the bacterial community composition was assessed from the soil cores taken in each ring as described above. Redundancy analysis (RDA) was used to explore associations between microbial community structures and environmental parameters and a Permutation test of redundancy analysis using 999 permutations was applied to evaluate their statistical significance [96].

Core microbiome ASVs of green and open inter-row soils were calculated by transforming the ASV counts to relative abundance with Microbiome version 1.8.0 [93]. Later, ASVs with a total relative abundance ≥ 0.01% and present in ≥ 85% samples were included as part of the core. For core genera estimation, ASVs were collapsed by genera and analyzed utilizing the settings described above.

Differential abundance of ASVs and genera from green inter-row soils was assessed by comparing the core bacterial compositions of each one utilizing the R package ALDEx2 1.22.0 [97]. First, ALDEx2 analysis was done by performing a centered log ratio (clr) transformation using as denominator the geometric mean abundance of all features and 128 Monte-Carlo instances; and then a Welch's t-test with a Benjamini–Hochberg correction with a threshold of < 0.05 was carried out. Features with absolute ALDEx effect sizes of > 0.8 and > 0.5 were considered to have a significantly greater and a moderate higher abundance, respectively.

### Microbe-microbe and microbiome-environmental parameters correlation analyses

Network analysis was performed using the core ASVs from aCO_2_ and eCO_2_ green inter-row soils, which showed an absolute ALDEx2 effect size > 0.5. Later, ASVs were analyzed utilizing a co-occurrence network with the R package Spiec-easi 1.1.1 [98], using the neighborhood selection method [99], a lambda path number of 100, a lambda minimum ratio of 10^–2^ and the Stability Approach to Regularization Selection (StARS) using its default settings. Subsequently, the network visualization was performed on Cytoscape 3.8.2 [100].

Similarly, Core genera co-occurrence from aCO_2_ and eCO_2_ green inter-rows samples were assessed with Spiec-easi 1.1.1 [98] and SPRING 1.0.4 [101] using genera with an absolute ALDEx2 effect size > 0.1 and using the neighborhood selection method [99], a lambda path number of 100, a lambda minimum ratio of 10^–1^ and the Stability Approach to Regularization Selection (StARS). Additionally, prior to SPRING partial correlation analysis, a modified central log ratio (mclr) transformation of the genera counts was performed.

Correlation analysis between green inter-rows ASVs and genera with environmental parameters was performed using ALDEx2 1.22.0 [97] and its “aldex.corr” function, utilizing Pearson's and Spearman's correlation coefficients, and the obtained p-values were corrected using the false discovery rate (FDR) method with a threshold of < 0.05.

### cDNA Quantitative PCR

The quantification of the 16S rRNA gene to estimate total bacterial abundance was performed following the protocol described by Kaplan et al. [80], but instead of DNA, cDNA products described above were used for the quantification. Likewise, the mRNA quantification of transcripts involved in the nitrogen cycle including nitrogen fixation (*nifH*), ammonia oxidation (*amoA*), nitrite reduction (*nirS, nirK*) and nitrous oxide reduction (*nosZ*) were performed using primers and amplification protocols described on Table 4 and expressed as percentage (%) of 16S rRNA copy numbers. Standards for the different genes were prepared from pure cultures or environmental clones as described by Kampmann et al. [102], and tenfold serial dilutions of the standards were used as templates, in triplicate, to determine the calibration curves. Total gene copy numbers of the standards were calculated according to Kampmann et al. [102]. All quantitative PCR (qPCR) was conducted in triplicate on a Rotor Gene Q (Qiagen, Hilden, Germany) by using Absolute qPCR SYBR Green Mix (ThermoFischer Scientific). Statistical comparisons were done with Kruskal–Wallis and Wilcoxon tests with the Benjamini & Hochberg adjustment method using R Package stats version 3.6.3.

**Table 4:** Primer sets and thermal profiles of transcripts for N cycle functional genes and 16S rRNA

qPCR target	Primer set	Thermal cycling profile	No. cycles	Reference
**16S RNA**	520F, 926R complemented	95° C/45 s, 60 °C/45 s, 72 °C/60 s, 84 °C/20 s	40	[78, 79]
***amoA***	amoA1_F, amoA2_R	95 °C/30 s, 59 °C/30 s, 72 °C/20 s, 80 °C/20 s	35	[103]
***nifH***	IGK3, DVV	95 °C/20 s, 55 °C/30 s, 72 °C/30 s, 84 °C/20 s	40	[58]
***nirK***	nirK876, nirK 5R	95 °C/20 s, 63 °C/25 s, 72 °C/60 s, 80 °C/20 s	40	[104, 105]
***nirS***	Cd3aF, R3cd	95 °C/20 s, 63 °C/25 s, 72 °C/60 s, 80 °C/20 s	40	[106, 107]
***nosZ***	nosZ2F, nosZ2R	95 °C/30 s, 63 °C/50 s, 72 °C/50 s, 80 °C/20 s	40	[108]

## Supplementary Information

**Additional file 1.** Elevated atmospheric CO_2_ concentrations caused a shift of themetabolically active microbiome in vineyard soil. **Table S1**, **Figure S1**-**S10**.

**Additional file 2:** **Table S2.** Chemical and soil respiration results from the Geisenheim VineyardFACE

**Additional file 3:Table S3.1.** Correlation of green inter-rows ASVs with environmental parameters using ALDEx2 with Pearson and Sperman correlation test and False Discovery Rate (FDR) for *p*-values correction. *r* = Pearson correlation coefficient.

## Data Availability

The authors declare that the data supporting the findings of this study are available within the article and its supplementary information. cDNA sequence data are available in the GenBank database under the accession number PRJNA680929.
