# Supplementary material for: Elevated atmospheric CO2 concentrations caused a shift of the metabolically active microbiome in vineyard soil
Source: BMC Microbiol. 2023 Feb 21;23:46. doi: 10.1186/s12866-023-02781-5 (PMC9942357; doi:10.1186/s12866-023-02781-5)
Supplement: Supplementary file 1 — Additional file 1. Elevated atmospheric CO2 concentrations caused a shift of themetabolically active microbiome in vineyard soil. Table S1, Figure S1-S10. [file 12866_2023_2781_MOESM1_ESM.docx]

**Supplementary material 1**

Elevated atmospheric CO_2_ concentrations caused a shift of the metabolically active microbiome in vineyard soil

David Rosado-Porto, Stefan Ratering, Yvette Wohlfahrt, Bellinda Schneider, Andrea Glatt, Sylvia Schnell

1. **Beta diversity dispersion of soil cores per ring**

For evaluation of the differences between the four replicate soil core samples within the six rings beta diversity dispersion was analysed, after centered log ratio transformation, creation of a distance matrix on an euclidian space and an ordination using Principal Components method.

-
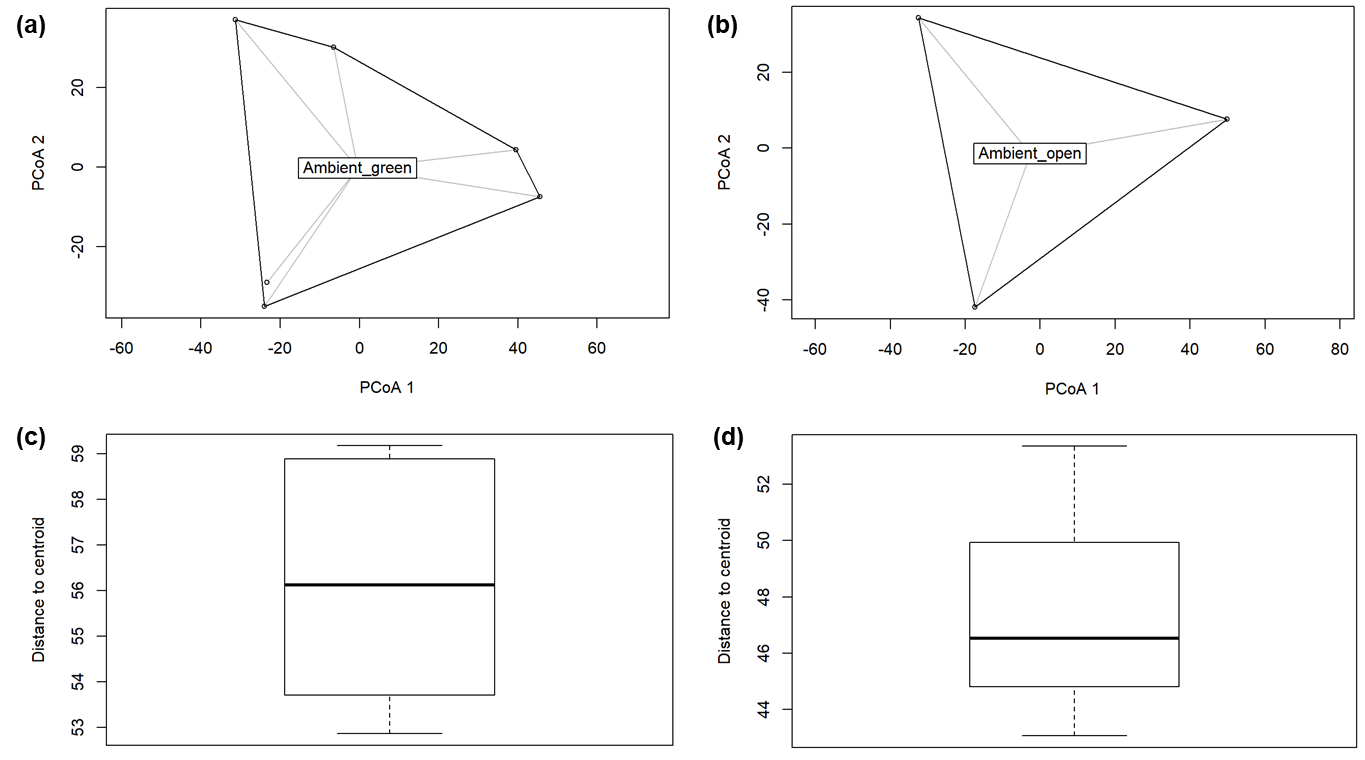
**Dispersion of soil cores from ring ambient CO_2_ A1 (Golf)**

PCA 2

PCA 2

PCA 1

PCA 1

**Figure S1.1 a)** Green inter-rows soil cores distance to the centroid on the Aitchison distance space. **b)** Open inter-rows soil cores distance to the centroid on the Aitchison distance space. **c)** Boxplot of the dispersion of green inter-rows soil cores to the centroid. **d)** Boxplot of the dispersion of open inter-rows soil cores to the centroid.

**Table S1.1** Principal coordinates analysis (PCA) results from green and open inter-row soils of Ring A1 from Geisenheim VineyardFACE.

| Soil core | Green inter-rows soil | |  | Open inter-rows soil | |
| --- | --- | --- | --- | --- | --- |
|  | PCA1 | PCA2 |  | PCA1 | PCA2 |
| 1 | -6.441 | 30.185 |  | 49.911 | 7.605 |
| 2 | -31.310 | 37.103 |  | -17.482 | -41.901 |
| 3 | -24.034 | -35.122 |  | -32.428 | 34.296 |
| 4 | -23.344 | -29.077 |  | ------ | ------ |
| 5 | 39.589 | 4.390 |  | ------ | ------ |
| 6 | 45.541 | -7.479 |  | ------ | ------ |
| Centroid | **-0.512** | **-0.223** |  | **-2.650** | **-1.463** |

- **Dispersion of soil cores from ring ambient CO_2_ A2 (Kilo)**

**
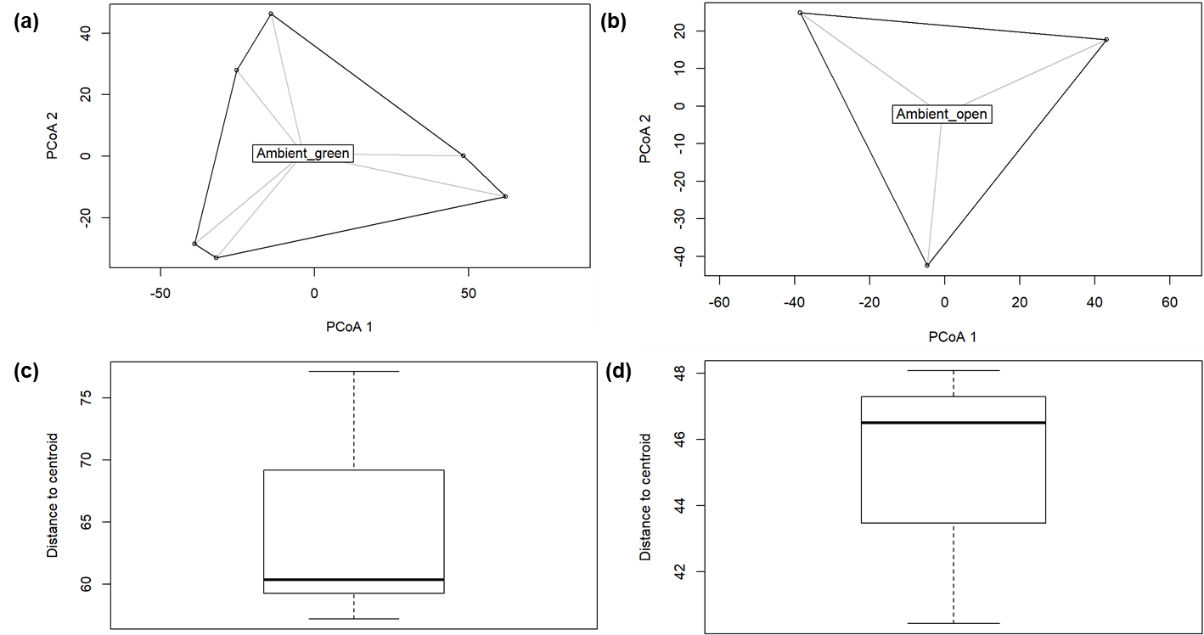
**

PCA 1

PCA 1

PCA 2

PCA 2

**Figure S1.2 a)** Green inter-rows soil cores distance to the centroid on the Aitchison distance space. **b)** Open inter-rows soil cores distance to the centroid on the Aitchison distance space. **c)** Boxplot of the dispersion of green inter-rows soil cores to the centroid. **d)** Boxplot of the dispersion of open inter-rows soil cores to the centroid.

**Table S1.2.** Principal coordinates analysis (PCA) results from green and open inter-row soils of Ring A2 from Geisenheim VineyardFACE.

| Soil core | Green inter-rows soil | |  | Open inter-rows soil | |
| --- | --- | --- | --- | --- | --- |
|  | PCA1 | PCA2 |  | PCA1 | PCA2 |
| 1 | -25.291 | 27.925 |  | 43.102 | 17.649 |
| 2 | -14.088 | 46.213 |  | -4.590 | -42.464 |
| 3 | -38.982 | -28.382 |  | -38.511 | 24.815 |
| 4 | -31.839 | -32.932 |  | ------ | ------ |
| 5 | 48.276 | 0.254 |  | ------ | ------ |
| 6 | 61.926 | -13.077 |  | ------ | ------ |
| Centroid | **-3.702** | **0.872** |  | **-0.674** | **-2.226** |

- **
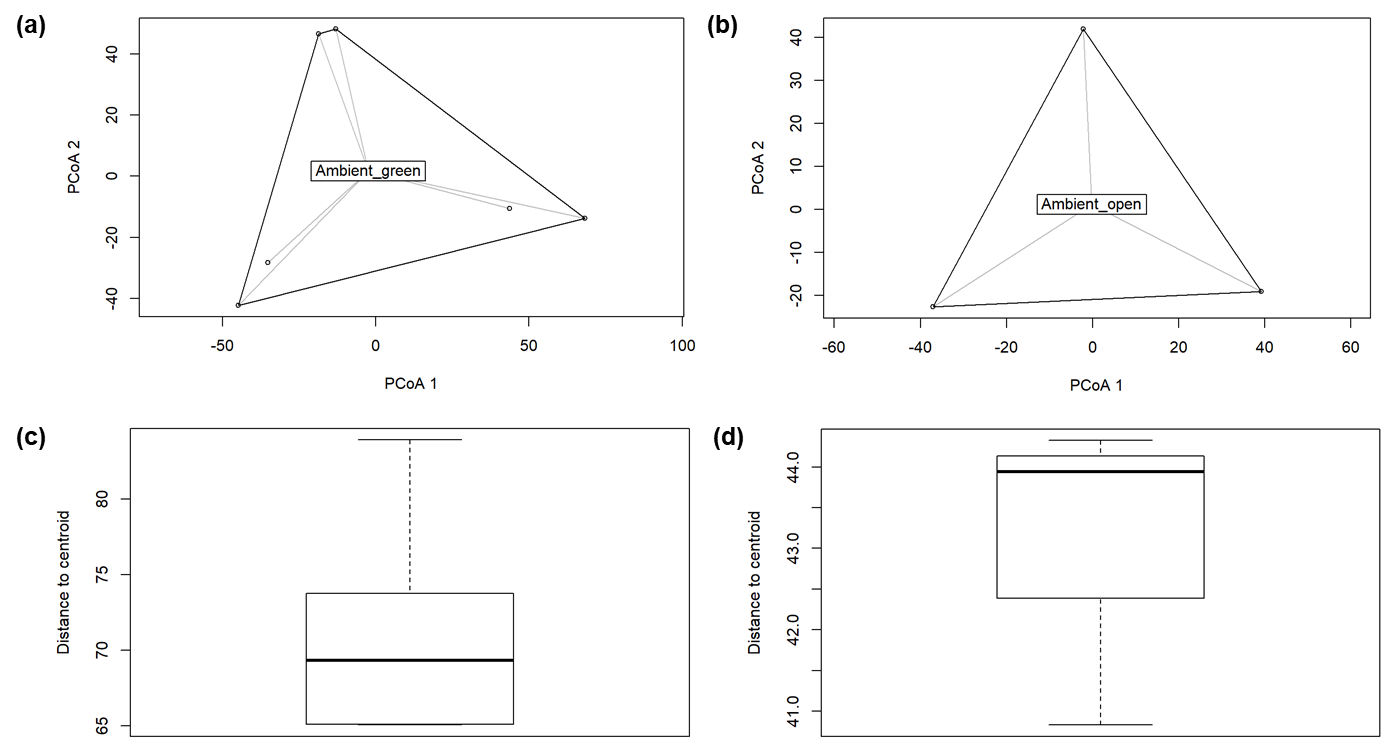
Dispersion of soil cores from ring ambient CO_2_ A3 (Lima)**

PCA 1

PCA 1

PCA 2

PCA 2

**Figure S1.3 a)** Green inter-rows soil cores distance to the centroid on the Aitchison distance space. **b)** Open inter-rows soil cores distance to the centroid on the Aitchison distance space. **c)** Boxplot of the dispersion of green inter-rows soil cores to the centroid. **d)** Boxplot of the dispersion of open inter-rows soil cores to the centroid.

**Table S1.3** Principal coordinates analysis (PCA) results from green and open inter-row soils of Ring A3 from Geisenheim VineyardFACE.

| Soil core | Green inter-rows soil | |  | Open inter-rows soil | |
| --- | --- | --- | --- | --- | --- |
|  | PCA1 | PCA2 |  | PCA1 | PCA2 |
| 1 | 43.672 | -10.460 |  | -2.148 | 41.864 |
| 2 | 68.222 | -13.681 |  | -37.117 | -22.698 |
| 3 | -13.111 | 48.050 |  | 39.265 | -19.166 |
| 4 | -18.684 | 46.585 |  | ------ | ------ |
| 5 | -35.219 | -28.264 |  | ------ | ------ |
| 6 | -44.879 | -42.229 |  | ------ | ------ |
| Centroid | **-2.526** | **1.708** |  | **-0.164** | **1.083** |

- **
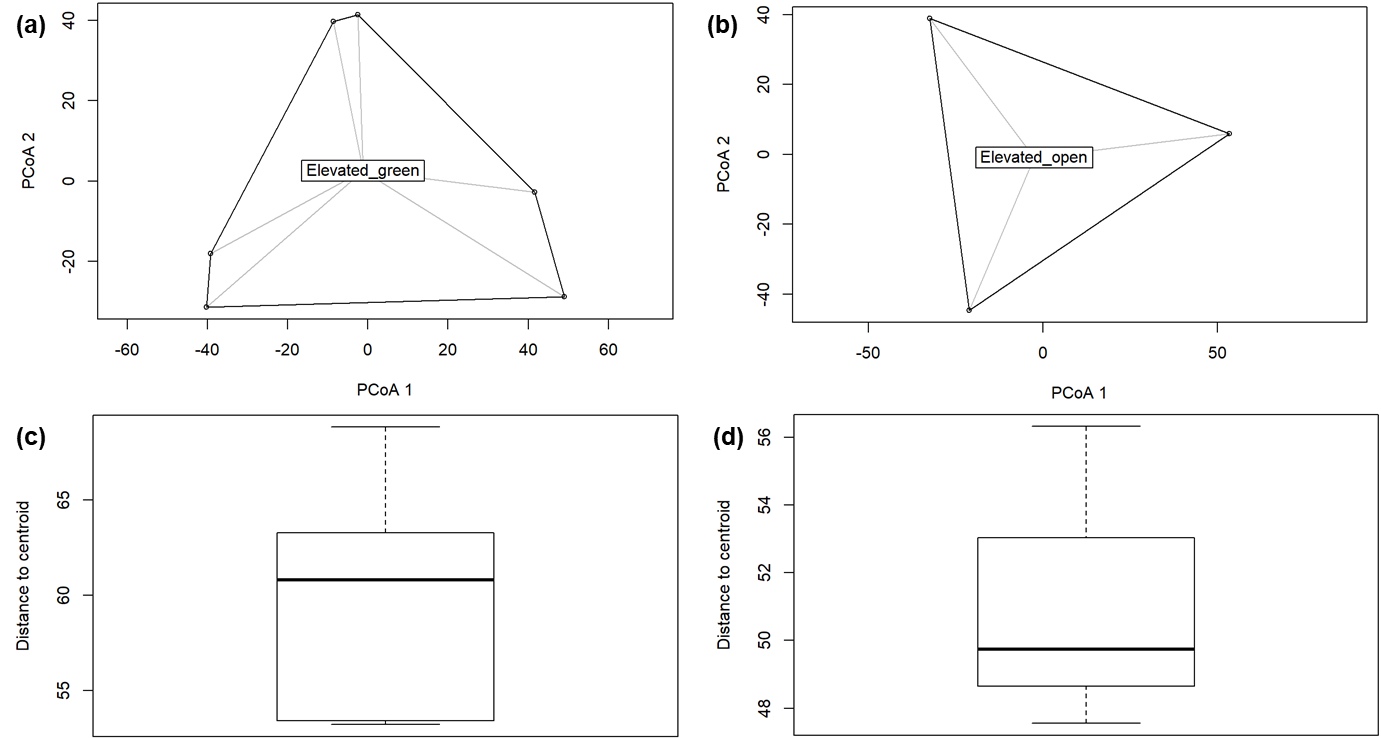
Dispersion of soil cores from ring elevated CO_2_ E1 (Delta)**

PCA 1

PCA 1

PCA 2

PCA 2

PCA 1

PCA 1

PCA 2

PCA 2

PCA 2

PCA 1

PCA 2

PCA 1

PCA 1

PCA 2

PCA 1

PCA 2

**Figure S1.4 a)** Green inter-rows soil cores distance to the centroid on the Aitchison distance space. **b)** Open inter-rows soil cores distance to the centroid on the Aitchison distance space. **c)** Boxplot of the dispersion of green inter-rows soil cores to the centroid. **d)** Boxplot of the dispersion of open inter-rows soil cores to the centroid.

**Table S1.4** Principal coordinates analysis (PCA) results from green and open inter-row soils of Ring E1 from Geisenheim VineyardFACE.

| Soil core | Green inter-rows soil | |  | Open inter-rows soil | |
| --- | --- | --- | --- | --- | --- |
|  | PCA1 | PCA2 |  | PCA1 | PCA2 |
| 1 | 41.611 | -2.763 |  | -32.333 | 38.834 |
| 2 | 49.002 | -28.857 |  | -21.110 | -44.681 |
| 3 | -2.514 | 41.388 |  | 53.443 | 5.846 |
| 4 | -8.617 | 39.772 |  | ------ | ------ |
| 5 | -39.204 | -18.061 |  | ------ | ------ |
| 6 | -40.277 | -31.478 |  | ------ | ------ |
| Centroid | **-1.257** | **2.545** |  | **-2.461** | **0.932** |

- **
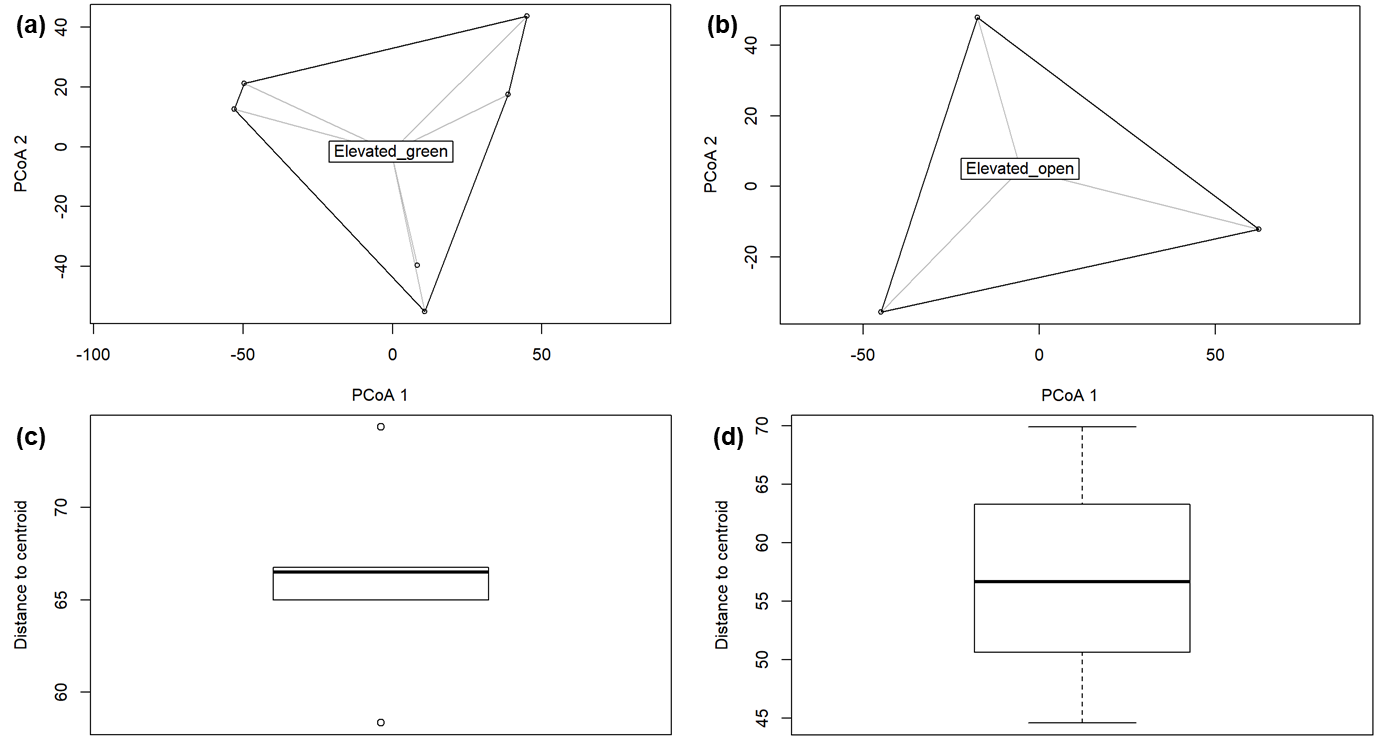
Dispersion of soil cores from ring elevated CO_2_ E2 (Bravo)**

PCA 2

PCA 1

PCA 1

PCA 2

**Figure S1.5 a)** Green inter-rows soil cores distance to the centroid on the Aitchison distance space. **b)** Open inter-rows soil cores distance to the centroid on the Aitchison distance space. **c)** Boxplot of the dispersion of green inter-rows soil cores to the centroid. **d)** Boxplot of the dispersion of open inter-rows soil cores to the centroid.

**Table S1.5** Principal coordinates analysis (PCA) results from green and open inter-row soils of Ring E2 from Geisenheim VineyardFACE.

| Soil core | Green inter-rows soil | |  | Open inter-rows soil | |
| --- | --- | --- | --- | --- | --- |
|  | PCA1 | PCA2 |  | PCA1 | PCA2 |
| 1 | -49.751 | 21.138 |  | -17.523 | 47.85949 |
| 2 | -53.041 | 12.496 |  | -44.846 | -35.66279 |
| 3 | 38.708 | 17.424 |  | 62.369 | -12.196 |
| 4 | 45.013 | 43.588 |  | ------ | ------ |
| 5 | 8.275 | -39.548 |  | ------ | ------ |
| 6 | 10.795 | -55.098 |  | ------ | ------ |
| Centroid | **-0.419** | **-1.611** |  | **-5.364** | **4.952** |

**
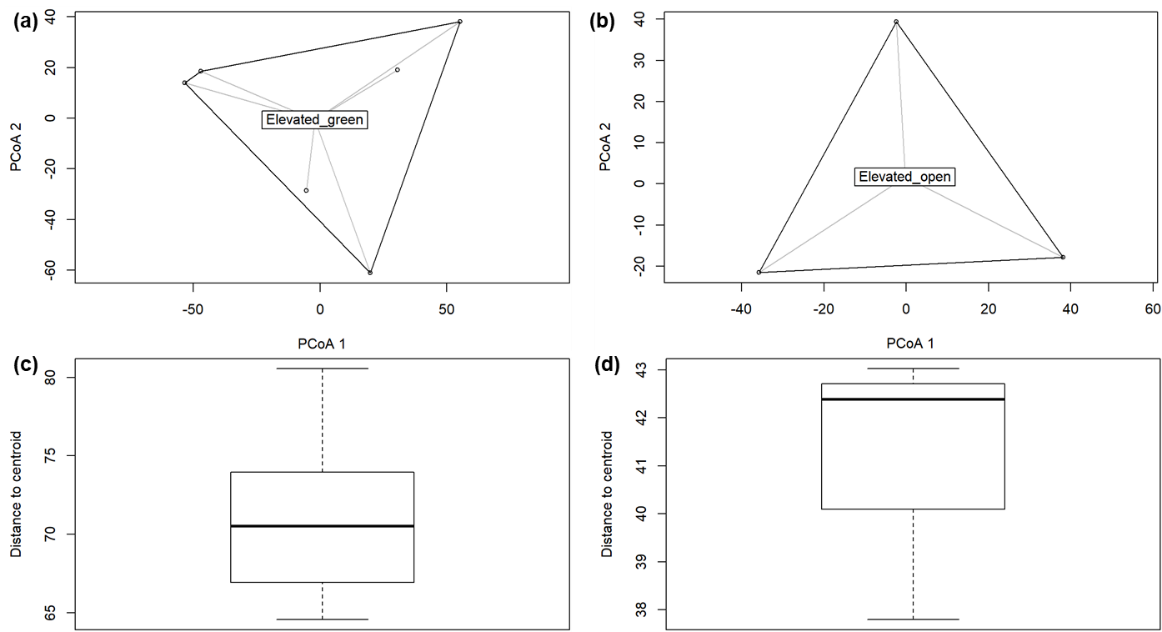
Dispersion of soil cores from ring elevated CO_2_ E3 (Echo)**

PCA 1

PCA 1

PCA 2

PCA 2

**Figure S1.6 a)** Green inter-rows soil cores distance to the centroid on the Aitchison distance space. **b)** Open inter-rows soil cores distance to the centroid on the Aitchison distance space. **c)** Boxplot of the dispersion of green inter-rows soil cores to the centroid. **d)** Boxplot of the dispersion of open inter-rows soil cores to the centroid.

**Table S1.6** Principal coordinates analysis (PCA) results from green and open inter-row soils of Ring E3 from Geisenheim VineyardFACE.

| Soil core | Green inter-rows soil | |  | Open inter-rows soil | |
| --- | --- | --- | --- | --- | --- |
|  | PCA1 | PCA2 |  | PCA1 | PCA2 |
| 1 | -53.4007 | 13.986 |  | -2.346 | 39.354 |
| 2 | -46.9905 | 18.613 |  | -35.773 | -21.551 |
| 3 | -5.329 | -28.636 |  | 38.119 | -17.802 |
| 4 | 19.905 | -61.046 |  | ------ | ------ |
| 5 | 30.547 | 19.044 |  | ------ | ------ |
| 6 | 55.269 | 38.037 |  | ------ | ------ |
| Centroid | **-2.074** | **-0.607** |  | **-0.273** | **1.616** |

**Conclusions**

The beta diversity results and its dispersion for each ring showed that the cores taken per ring are different enough to be at different positions on the Aitchison space. Furthermore, when analyzing the dispersion and their distance to the centroids, all of them showed a great level of dispersion, which validated that the soil cores are sufficiently different not to be considered as technical replicates, but single samples.

1.
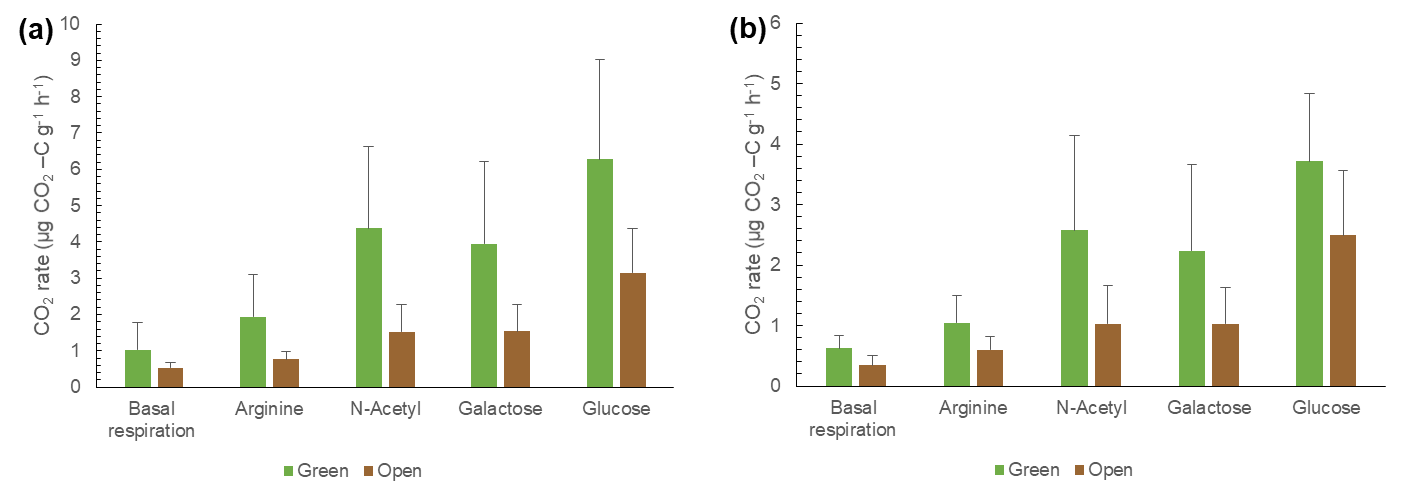
**Soil respiration comparison of green and open inter-rows from Geisenheim VineyardFACE**

**Fig. S1.7**. Soil microbial respiration expressed as CO_2_ production rate under the addition of different carbon substrates. (a) Soil from elevated CO_2_ rings, (b) soil from ambient CO_2_ rings. Error bars are expressed as variance of mean values (n=18).

**Table S1.7** Soil microbial respiration under the addition of different carbon substrates expressed as CO_2_ rate (µg CO_2_-C g^-1^ h^-1^) from ambient and elevated CO_2_ rings. Error is expressed as variance of mean values (n=18). p-values significance codes from t-test for samples with equal variances: 0.00001 ‘****’ ,0.0001 ‘***’ , 0.001 ‘**’ , 0.01 ‘*’ . Fold changes compare green rows/open inter-rows.

| CO_2_ conditions | Soil respiration | Green inter-rows | Open inter-rows | p-value | Fold change |
| --- | --- | --- | --- | --- | --- |
| Ambient | Basal respiration | 0.63 ± 0.21 | 0.34 ± 0.16 | 0.001*** | 1.81 |
|  | Arginine | 1.05 ± 0.45 | 0.60 ± 0.20 | 0.001*** | 1.74 |
|  | N-Acetyl glucosamine | 2.57 ± 1.56 | 1.03 ± 0.62 | 0.001*** | 2.50 |
|  | Galactose | 2.24 ± 1.41 | 1.03 ± 0.59 | 0.004** | 2.18 |
|  | Glucose | 3.71 ± 1.11 | 2.49 ± 1.06 | 0.015* | 1.49 |
| Elevated | Basal respiration | 1.03 ± 0.75 | 0.53 ± 0.14 | 0.013* | 1.95 |
|  | Arginine | 1.94 ± 1.16 | 0.77 ± 0.21 | 0.0005*** | 2.51 |
|  | N-Acetyl | 4.37 ± 2.25 | 1.52 ± 0.75 | 0.00006**** | 2.87 |
|  | Galactose | 3.95 ± 2.26 | 1.54 ± 0.73 | 0.0004*** | 2.57 |
|  | Glucose | 6.27 ± 2.76 | 3.14 ±1.21 | 0.0007*** | 2.00 |

**Table S1.8.** Soil microbial respiration under the addition of different carbon substrates. Respiration expressed as CO_2_ rate (µg CO_2_-C g^-1^ h^-1^) from green inter-rows from ambient and elevated CO_2_ rings, and from open inter-rows from ambient and elevated CO_2_ rings. Error is expressed as variance of mean values (n=18). P-values significance codes are from a t-test for samples with equal variances. Significance codes: ** p<0.01, * p<0.05.

| Rows | Soil respiration | Ambient | Elevated | p-value | Fold Change |
| --- | --- | --- | --- | --- | --- |
| Green | Basal respiration | 0.62 ± 0.04 | 1.03 ± 0.57 | 0.040* | 1.65 |
|  | Arginine | 1.05 ± 0.20 | 1.93 ± 1.35 | 0.006** | 1.85 |
|  | N-Acetyl glucosamine | 2.57 ± 1.56 | 4.37 ± 2.25 | 0.009** | 1.70 |
|  | Galactose | 2.23 ± 1.41 | 3.95 ± 2.26 | 0.01* | 1.77 |
|  | Glucose | 3.71 ± 1.11 | 6.33 ± 2.90 | 0.003** | 1.69 |
| Open | Basal respiration | 0.34 ± 0.02 | 0.52 ± 0.02 | 0.02* | 1.53 |
|  | Arginine | 0.6 ± 0.04 | 0.77 ± 0.04 | 0.11 | 1.28 |
|  | N-Acetyl glucosamine | 1.03 ± 0.39 | 1.52 ± 0.56 | 0.15 | 1.48 |
|  | Galactose | 1.02 ± 0.35 | 1.53 ± 0.53 | 0.12 | 1.50 |
|  | Glucose | 2.49 ± 1.14 | 3.14 ± 1.47 | 0.24 | 1.26 |

1. **Taxa with highest relative abundance**

**Fig. S1.8**. Relative abundance barplot of the ten most abundant bacterial phyla.

**Fig. S1.9**. Relative abundance barplot of the ten most abundant bacterial classes.

**Fig. S1.10**. Relative abundance barplot of the ten most abundant bacterial families.
